# A New Electrochemical Platform for Dasatinib Anticancer Drug Sensing Using Fe_3_O_4_-SWCNTs/Ionic Liquid Paste Sensor

**DOI:** 10.3390/mi12040437

**Published:** 2021-04-14

**Authors:** Ali Moghaddam, Hassan Ali Zamani, Hassan Karimi-Maleh

**Affiliations:** 1 Department of Applied Chemistry, Mashhad Branch, Islamic Azad University, Mashhad 9187147578, Iran; ali.moghaddam1370@gmail.com; 2 Laboratory of Nanotechnology, Department of Chemical Engineering and Energy, Quchan University of Technology, Quchan 9477177870, Iran

**Keywords:** dasatinib, Fe_3_O_4_-SWCNTs nanocomposite, ionic liquid, sensor

## Abstract

A new electrochemical platform was suggested for the sensing of the dasatinib (DA) anticancer drug based on paste electrode modification (PE) amplified with Fe_3_O_4_-SWCNTs nanocomposite and 1-hexyl-3-methylimidazolium tetrafluoroborate (mim-BF_4_^−^). The new platform showed a linear dynamic range from 0.001–220 µM with a detection limit of 0.7 nM to determine DA at optimal condition. Electrochemical investigation showed that the redox reaction of DA is relative to changing the pH of solution. Moreover, Fe_3_O_4_-SWCNTs/mim-BF_4_^−^/PE has improved the oxidation current of DA about 5.58 times which reduced its oxidation potential by about 120 mV at optimal condition. In the final step, Fe_3_O_4_-SWCNTs/mim-BF_4_^−^/PE was used as an analytical platform to determine the DA in tablets and a dextrose saline spike sample, and the results showed recovery data 99.58–103.6% which confirm the powerful ability of the sensor as an analytical tool to determine the DA in real samples.

## 1. Introduction

Cancer is a major global problem and the cause of many deaths. Breast cancer is one of the most common and deadly cancers in women, and due to statistics, one in eight American women will develop this cancer during their lifetime [1]. Furthermore, use of anticancer drugs has significantly grown, and various anticancer drugs are used for chemotherapy [2,3,4,5]. With the brand name Sprycel, dasatinib (DA) is usually used to treat prostate and breast cancers [6,7]. Taking too much DA can cause many side effects, such as bleeding, rash, and diarrhea in patients. Due to the high risk of using this drug, such as the increased risk of an infection that is relative to a drop in white blood cells, controlling the patient while taking this drug by a doctor or nurse is very important and necessary; however, using the appropriate judgment requires surveying its effect on the patient’s body which is impossible without using analytical methods [8,9]. Moreover, analytical methods were accepted as an integral part of medical and patient treatment in most treatment systems in different countries [10,11,12]. Many analytical methods were suggested to determine anticancer drugs such as DA in biological and pharmaceutical samples such as high-performance liquid chromatography (HPLC) [13,14,15,16], spectroscopic strategies [17], liquid chromatography mass spectrometry (LC-MS) [18] and electrochemical sensors [19,20,21]. Although chromatographic methods have long been used for this purpose, their use was hampered by the disadvantages such as the use of toxic solvents, the need for a skilled operator, and the inability to convert portable kits [22]. Furthermore, research has ensued to provide a fast, inexpensive, high-performance method to measure the important biological compounds [23,24,25,26,27,28,29].

Electrochemical methods are a sensitive and powerful approach to determine a wide range of biological and pharmaceutical compounds, especially anticancer drugs in biological conditions [30,31,32,33,34,35,36]. The wide range of the applications of electrochemical sensors is relative to easy modification of electrochemical sensors [37,38,39,40,41,42]. In this regard, and due to the purpose of measurement, various modifications can be made to electrochemical sensors, increasing the sensitivity and selectivity of measurement [43,44,45,46,47].

Nanomaterials are a very important and useful group of materials which have created a dramatic change in the world of science [48,49,50,51,52,53,54,55,56]. Nanomaterials have created unique properties and dramatically changed various analytical treatment techniques [57,58,59,60]. One of the unique features of nanomaterials is their high electrical conductivity which has created favorable conditions for the design of sensitive electrochemical sensors [61,62,63]. Among these, high electrical conductivity carbon/metal nanocomposites were proposed in recent years to manufacture various electrochemical sensors [64]. In the literature, Fe_3_O_4_ nanoparticle and carbon nanotubes showed good catalytic activity to fabricate electrochemical sensors. For example, Fang et al. reported a gold electrode amplified with Fe_3_O_4_ nanoparticle as an electrochemical sensor to determine the dopamine. Reported results showed good catalytic activity of Fe_3_O_4_ nanoparticles and confirmed that they are suitable for the fabrication of modified sensors [65]. On the other hand, many research and review papers confirmed the powerful ability of carbon nanotubes (SWCNTs or MWCNTs) as electrocatalysts for the fabrication of electrochemical sensors [66,67,68]. Based on this, it was predicted that iron oxide/carbon nanotube nanocomposite would show a more synergistic effect to prepare the electrochemical sensors. For example, Abbasghorbani reported the application of Fe_3_O_4_-SWCNTs nanocomposite as a conductive mediator to modify the epirubicin anticancer sensor with a detection limit of 7.0 nM [69]. Based on the presented materials, it seems that the design and construction of an analytical sensor with the ability to measure small amounts of DA can be useful to evaluate the effectiveness of this anticancer drug. For this purpose, Fe_3_O_4_-SWCNTs/1-hexyl-3-methylimidazolium tetrafluoroborate (mim-BF_4_^−)^/based on paste electrode modification (PE) is made with high sensitivity and a suitable detection limit for measuring DA, and the results obtained confirm its high capability in measuring real samples. Two-fold amplification of paste electrode with Fe_3_O_4_-SWCNTs and mim-BF_4_^−^ created a highly conductive sensor which could be detected dasatnib in nanomolar level (0.7 nM). This value of detection limit is comparable and better than the previous detection limit reported by electrochemical sensors for the determination of dasatinib (Table 1).

## 2. Materials and Methods

### 2.1. Materials and Synthesis Procedure

Dasatinib, SWCNTs-COOH, 1-hexyl-3-methylimidazolium tetrafluoroborate, graphite powder, ferric chloride, sodium hydroxide, phosphoric acid, iron (I) sulfate were purchased in analytical grade from Merck and Sigma-Aldrich Companies. The stock solution of dasatinib (0.01 M) was prepared by dissolving 0.244 g dasatinib into 50 mL ethanol/water (1:1) solution and ultrasonication for 30 min at room temperature. The chemical precipitation strategy described by Abbasghorbani was used for the synthesis of Fe_3_O_4_-SWCNTs nanocomposite [69]. For this goal, iron (II) sulfate and iron trichloride solutions with ration 2:1 were prepared into a graduated beaker containing 150 mL distilled water and stirred for 30 min. In continuous, 1.0 g SWCNTs-COOH + sodium hydroxide 3.0 M were added to the graduated beaker under nitrogen gas. The solid sample was filtered and then dried at a temperature of 150 °C for 16 h.

Fe_3_O_4_-SWCNTs/mim-BF_4_^−^/PE was organized using a mixing composition containing 60 mg Fe_3_O_4_-SWCNTs nanocomposite + 940 mg graphite powder in the presence of 10 drops of paraffin oil and 2 drops of mim-BF_4_^−^ for 3 h using a mortar and pestle. The prepared paste was added to the end of a glass tube with a diameter of 3 mm.

### 2.2. Apparatus

A potentiostat/galvanostat (Ivium-Vertex Company, Eindhoven, Netherlands), a machine connected with an electrochemical cell (Azar electrode) was designed for current-voltage (I-V) investigation in this research work. Pt wire (Azar Electrode Company, Urmia, Iran), Fe_3_O_4_-SWCNTs/mim-BF_4_^−^/PE, and Ag/AgCl/KCl_sat_ (reference electrode) Azar Electrode Company were used for the recording of I-V signals. A transmission electron microscope (TEM) image was recorded by a Zeiss-EM10C-100 KV (Germany) for morphological investigation.

### 2.3. Real Sample

The dasatinib tablet (80.0 mg dose of the drug per tablet) and dextrose saline were selected as real samples. Five tablets were powdered in mortar and pestle. Then, powdered tablets were dissolved in ethanol/water (1:1) solution and ultrasonication for 30 min at room temperature. After filtration, 5 mL of solution was diluted with 5 mL phosphate buffer solution pH = 6.0 and used for real sample analysis using the standard addition method. On the other hand, dextrose saline was spiked with different DA concentrations and directly used for real sample analysis of the anticancer drugs. I-V signals were recorded for cyclic voltammetric investigation in the potential range 0.3–0.9 V. I–V signals were recorded for square wave voltammetric investigation in the potential range 0.35–0.85 V with a frequency 10 Hz.

## 3. Results and Discussion

### 3.1. Electrochemical Behaviour of Dasatinib

**Figure 1:**
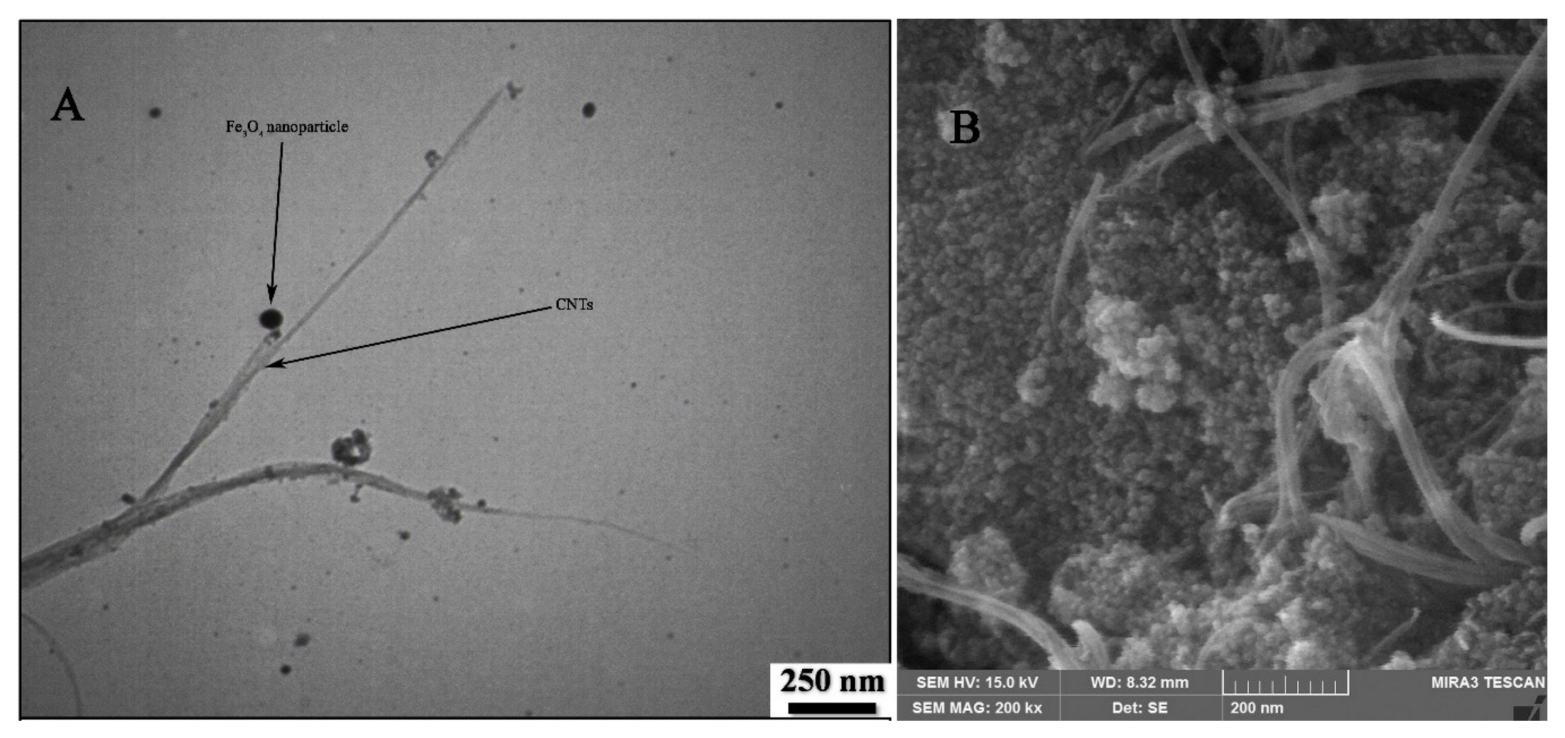
(**A**) Transmission electron microscope (TEM) and (**B**) field-emission scanning electron microscope (FESEM) images of Fe_3_O_4_-SWCNTs nanocomposite.

TEM and field-emission scanning electron microscopy (FESEM) images of Fe_3_O_4_-SWCNTs are shown in Figure 1A,B and the results clearly confirm the presence of Fe_3_O_4_ nanoparticles with spherical shape and good distribution at the surface of single-wall carbon nanotubes. Besides, EDS analysis showed the presence of Fe (29.53% *w*/*w*), O (33.77% *w*/*w*), and C (36.7 % *w*/*w*) elements that confirm good purity of Fe_3_O_4_-SWCNTs nanocomposite.

**Figure 2:**
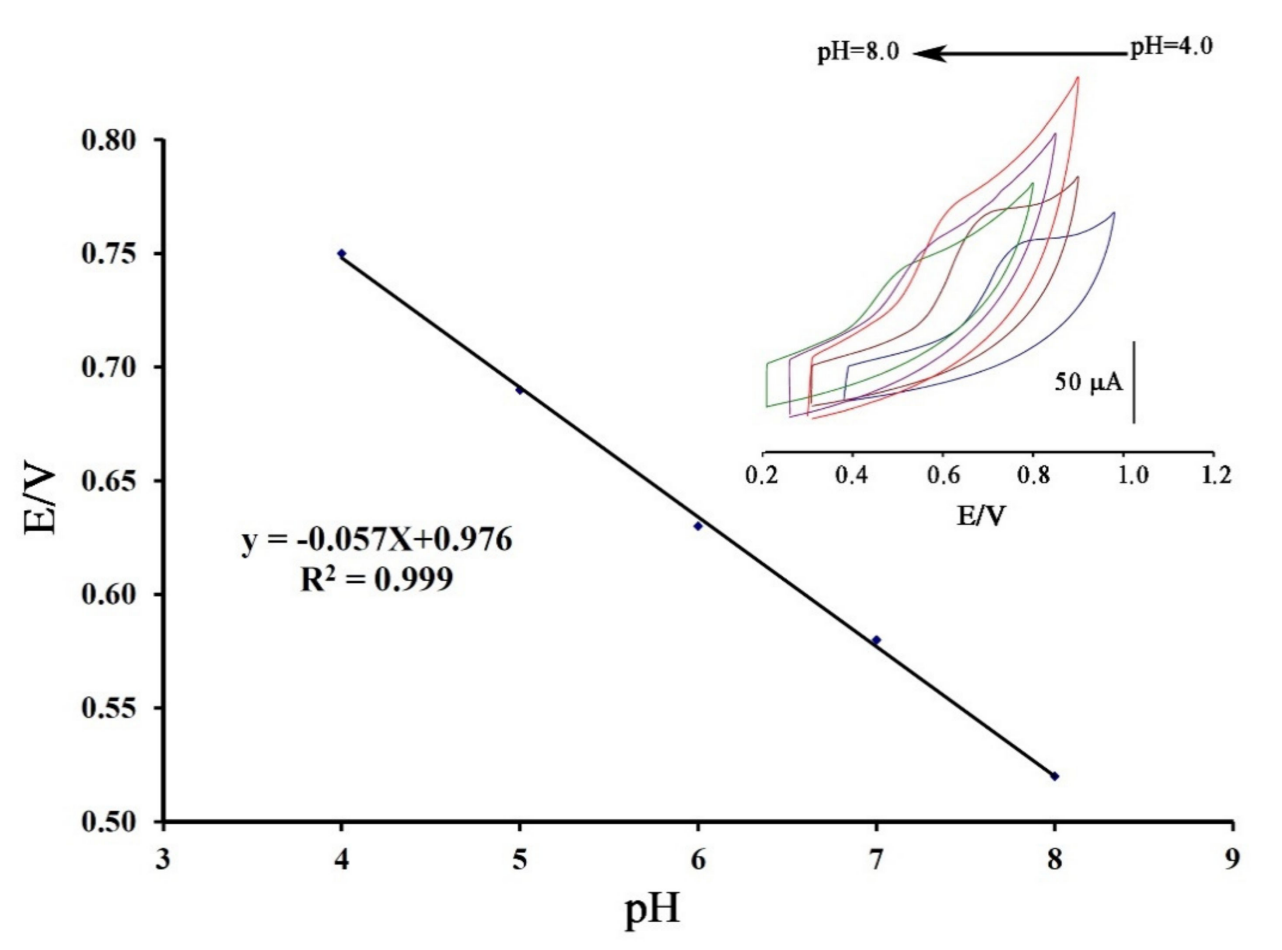
E-pH plot relative to electrooxidation of dasatinib (DA) at the surface of Fe_3_O_4_-SWCNTs/mim-BF_4_^−^/PE in different pH range. Inset: cyclic voltammograms of DA at the surface of Fe_3_O_4_-SWCNTs/mim-BF_4_^−^/PE in the different pH ranges with scan rate 100 mV/s.

**Scheme 1:**
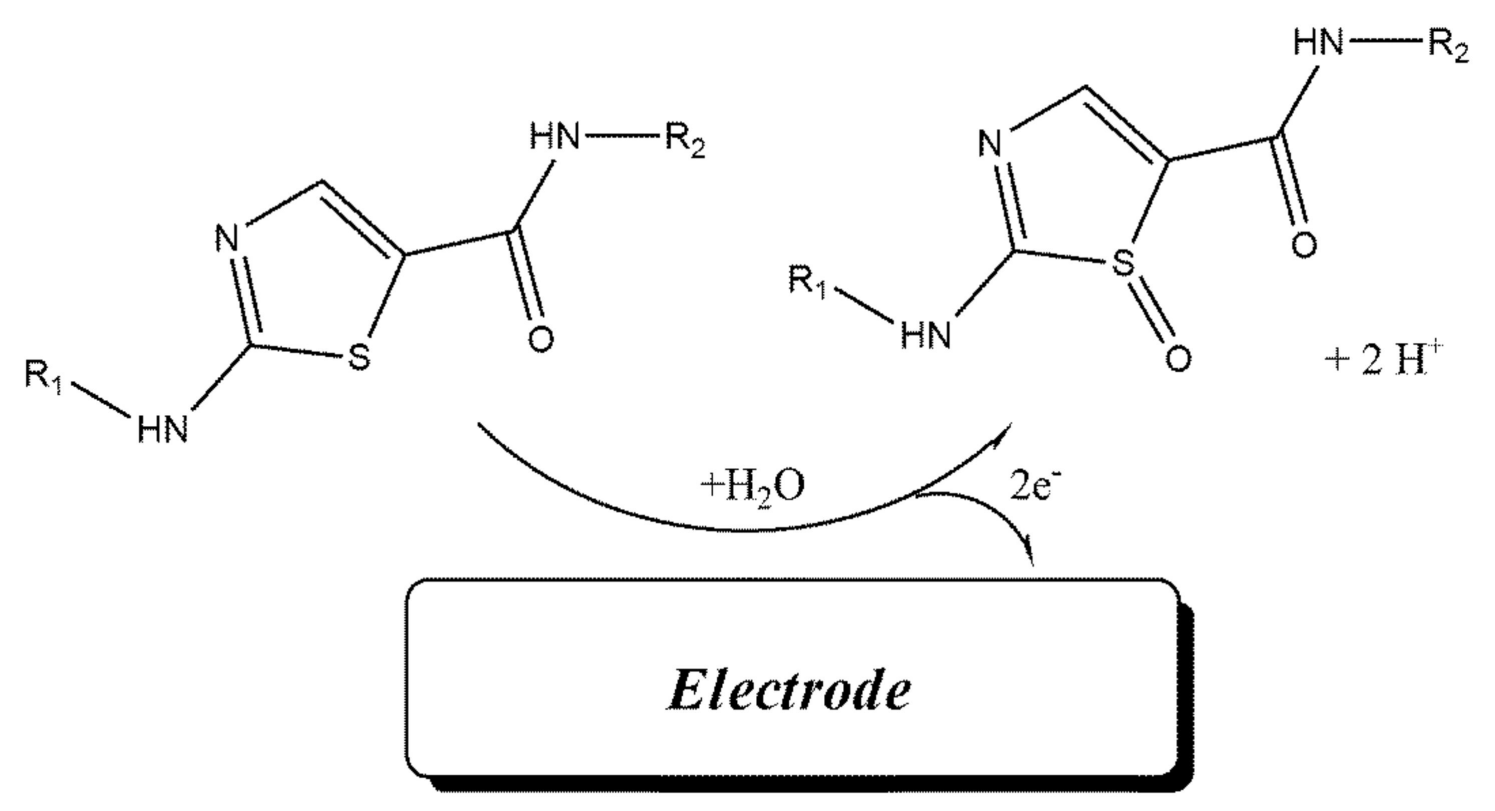
Electro-oxidation mechanism of dasatinib.

Cyclic voltammograms of DA were recorded at surface of Fe_3_O_4_-SWCNTs/mim-BF_4_^−^/PE in the different pH range (4.0 < pH < 8.0) (Figure 2 inset). The negative change in the oxidation potential of dasatinib with moving pH = 4.0 to pH = 8.0 confirms proton presence in redox reaction of DA. The linear relation between the oxidation potential of dasatinib anticancer drug vs. pH with Equation E = 0.057 pH + 0.976 (R^2^ = 0.999); suggests the presence of equal value of electron and proton in redox reaction of DA (Scheme 1). Moreover, maximum sensitivity for the DA signal was observed at pH = 6.0, and this pH was used for future investigation.

**Figure 3:**
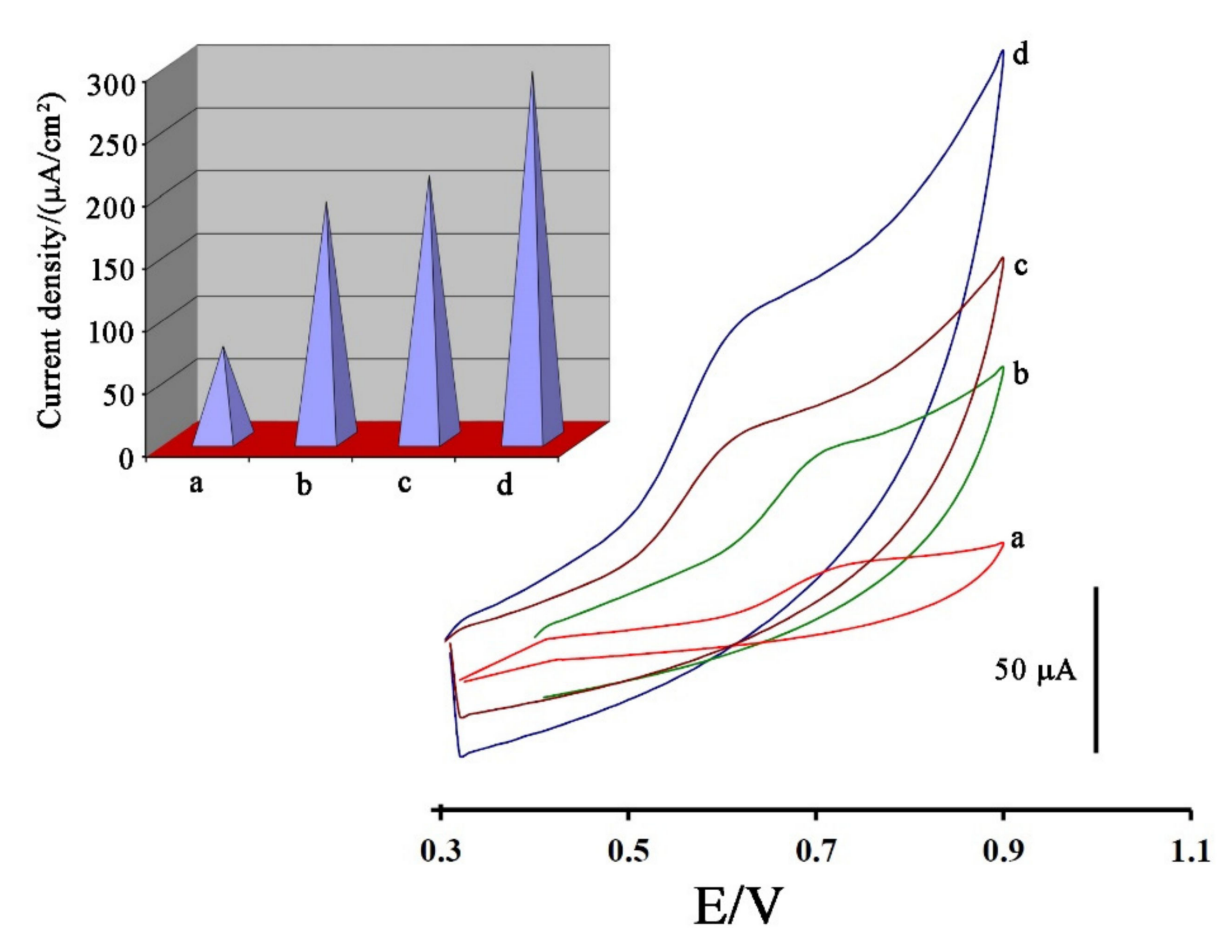
Cyclic voltammograms of 500 µM dasatinib anticancer drug at the surface of (**a**) carbon paste electrode, (**b**) Fe_3_O_4_-SWCNTs/PE, (**c**) mim-BF4^−^/PE and (**d**) Fe_3_O_4_-SWCNTs/mim-BF_4_^−^/PE. Inset: current density data relative to different electrodes. Conditions, pH = 6.0 and scan rate 100 mV/s.

In the next step, and for investigating Fe_3_O_4_-SWCNTs nanocomposite and mim-BF_4_^−^ in the fabrication of electrodes, the cyclic voltammograms of 500 µM DA were recorded at the surface of the carbon paste electrode (Figure 3 curve a), Fe_3_O_4_-SWCNTs/PE (Figure 3 curve b), mim-BF_4_^−^/PE (Figure 3 curve c) and Fe_3_O_4_-SWCNTs/mim-BF_4_^−^/PE (Figure 3 curve d), respectively. Due to the cyclic voltammograms, oxidation currents 20.47 µA, 56.128 µA, 54.32 µA, and 93.7 µA and oxidation potentials 740, 700, 630, and 620 mV were detected for the oxidation of DA at the surface of the carbon paste electrode, Fe_3_O_4_-SWCNTs/PE, mim-BF_4_^−^/PE and Fe_3_O_4_-SWCNTs/mim-BF_4_^−^/PE, respectively.

By comparing the results obtained, it can be understood that the presence of Fe_3_O_4_-SWCNTs nanocomposite and mim-BF_4_^−^ alone can improve the oxidation signal of DA. In addition, by two-fold amplification of the paste electrode with Fe_3_O_4_-SWCNTs nanocomposite and mim-BF_4_^−^, the oxidation current of dasatinib anticancer drug was improved about 5.58 times and oxidation potential of drug was decreased about 120 mV compare to unmodified electrode. These points confirms high conductivity of the suggested sensor for sensing of dasatinib in this work. For more investigation, the active surface area of electrodes was determined by the standard method ([Fe(CN)_6_]^3−/4−^ + 0.1 M KCl) and Randles–Sevcik Equation (1):

(1) $$I=2.69\times 10^{5}\;n^{3/2}A\;D^{1/2}\;v^{1/2}\;C\;$$

Using recorded signals and the Randles–Sevcik equation, the active surface area of carbon paste electrode, Fe_3_O_4_-SWCNTs/PE, mim-BF_4_^−^/PE and Fe_3_O_4_-SWCNTs/mim-BF_4_^−^/PE were determined about 0.274 cm^2^, 0.295 cm^2^, 0.305 cm^2^ and 0.319 cm^2^, respectively. In addition, current density curves are shown in Figure 3 inset. Based on recorded curves, it concluded that the presence of Fe_3_O_4_-SWCNTs nanocomposite and mim-BF_4_^−^ could be increased the conductivity of paste electrode and creating a good condition for sensing the dasatinib anticancer drug.

**Figure 4:**
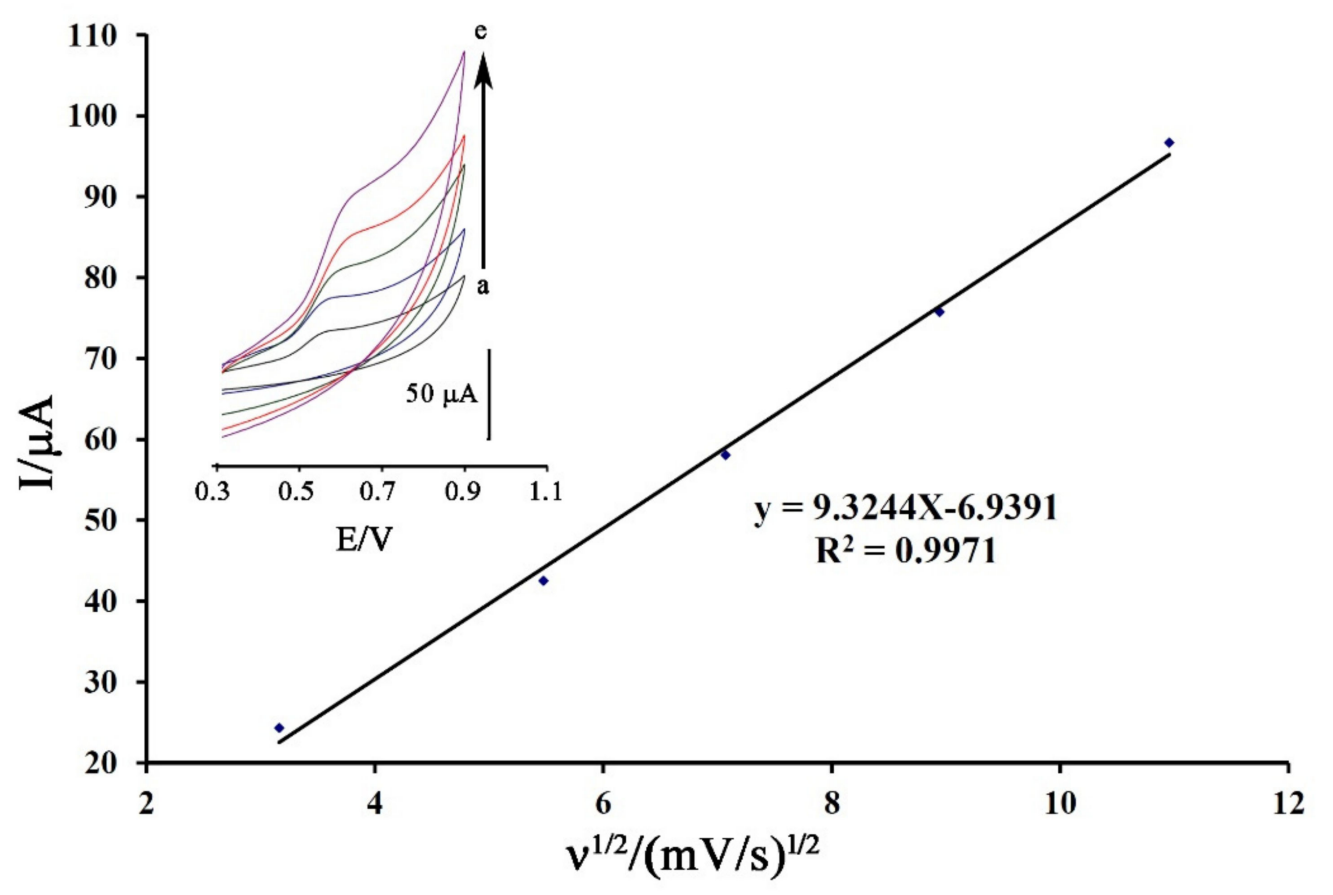
Plot of I-ν^1/2^ for electro-oxidation of DA at surface of Fe_3_O_4_-SWCNTs/mim-BF_4_^−^/PE using scan rates (**a**) 10; (**b**) 30; (**c**) 50; (**d**) 80 and (**e**) 120 mV/s. Condition, pH = 6.0.

The cyclic voltammogram of DA was recorded at the surface of Fe_3_O_4_-SWCNTs/mim-BF_4_^−^/PE in the different scan rates for the investigation of moving mechanism of dasatinib anticancer drug into electrode surface (Figure 4 inset). The oxidation current of DA showed a linear relation with *ν*^1/2^ (equation *I* = 9.3244 *ν*^1/2^−6.9391 (R^2^ = 0.9971)) that confirmed a diffusion process [72,73,74] for moving of dasatinib anticancer drug from solution to the electrode surface for redox reaction process.

**Figure 5:**
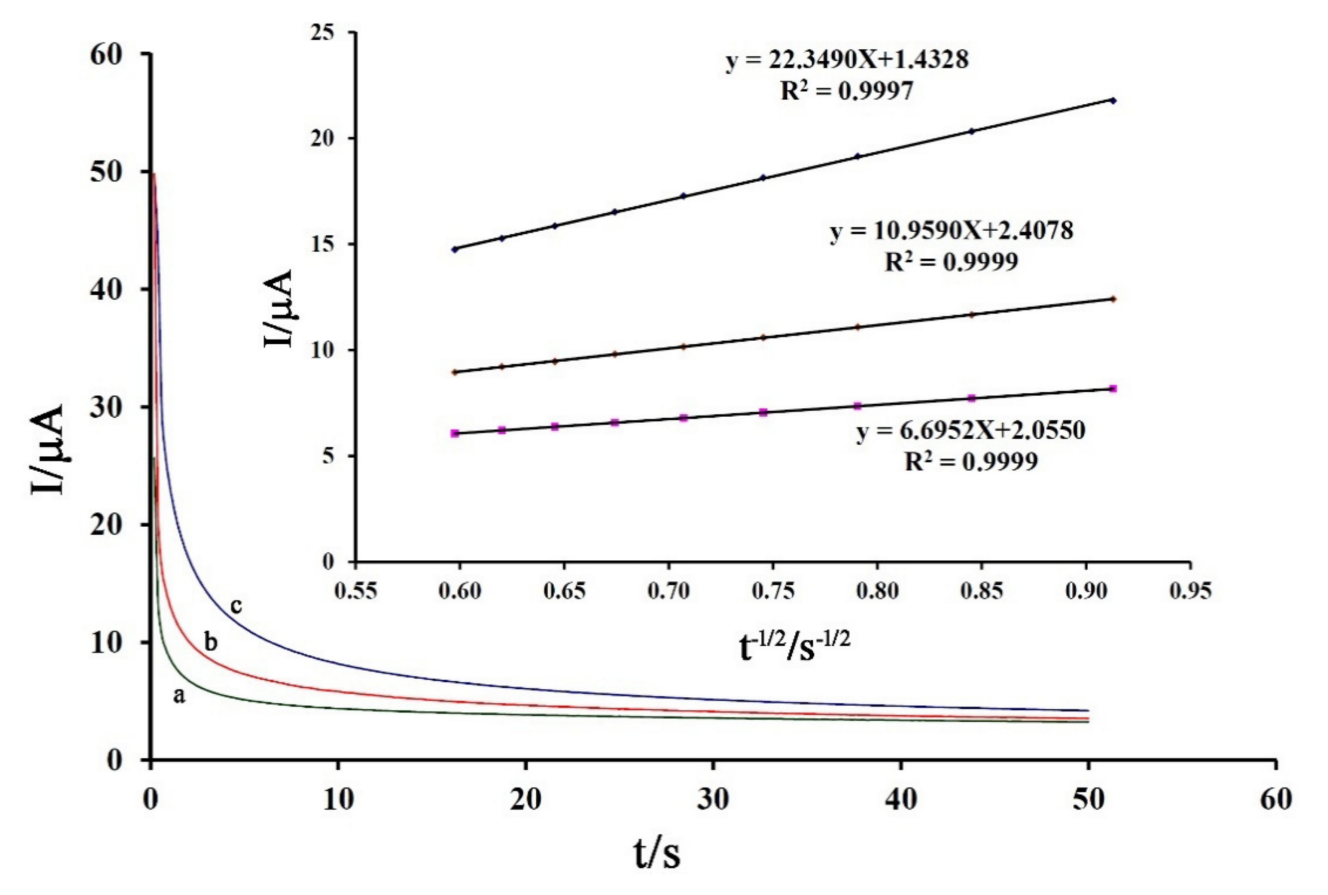
Chronoamperograms obtained at Fe_3_O_4_-SWCNTs/mim-BF_4_^−^/PE in the presence of (**a**) 300 µM, (**b**) 400 µM, and (**c**) 500 µM DA. (**Inset**) Dependence of I on the t^−1/2^ derived from DA signals. Condition, pH = 6.0 and applied potential of 750 mV.

After confirmation of the diffusion process for electro-oxidation of the drug, the diffusion coefficient (D) was obtained by chronoamperometric studies with an applied potential of 750 mV in the presence of 300 μM, 400 μM, and 500 μM of DA (Figure 5). Using recorded Cottrell’s plots and related slopes, the value of the diffusion coefficient was calculated to be about 3.57 × 10^−6^ cm^2^/s (Figure 5 inset).

Stability is one of the main factors of a new sensor to determine the biological compounds for long duration analysis. Therefore, the stability of Fe_3_O_4_-SWCNTs/mim-BF_4_^−^/PE for the determination of 20.0 µM DA was investigated in this step. For this investigation, a cyclic voltammogram of 20.0 µM DA was recorded at a period time 60 days. The oxidation current of 20.0 µM DA decreased by about 92% of the initial signal after 60 days, and the drug’s oxidation potential remained constant. This point confirms that Fe_3_O_4_-SWCNTs/mim-BF_4_^−^/PE has good stability for the sensing of DA for two months.

**Figure 6:**
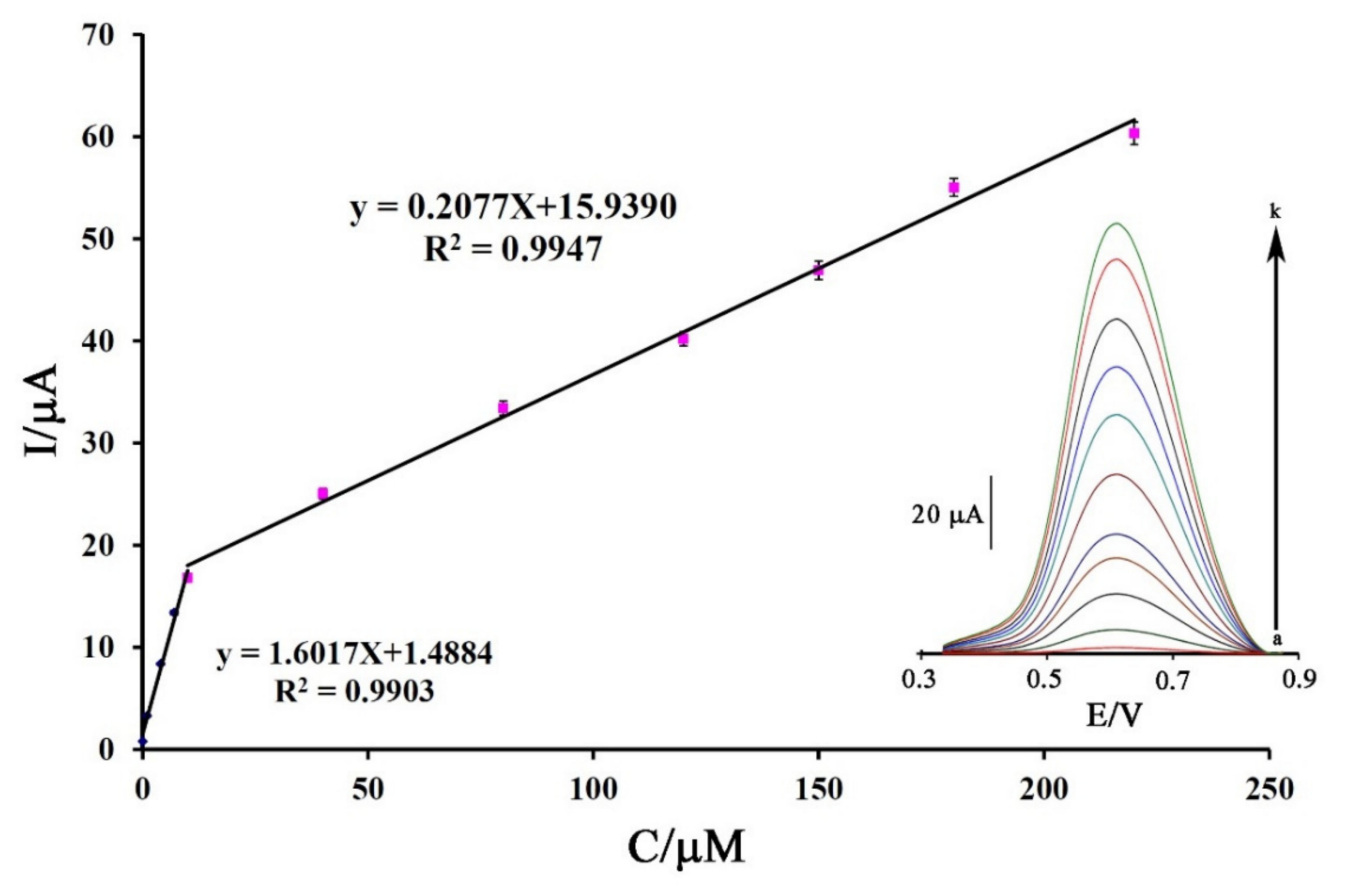
Plot of Current-concentration for electro-oxidation of DA at the surface of Fe_3_O_4_-SWCNTs/mim-BF_4_^−^/PE. (**Inset**) SW voltammograms of DA at the surface of Fe_3_O_4_-SWCNTs/mim-BF_4_^−^/PE in the concentration range 0.001–220 µM. Condition, pH = 6.0 and *n* = 3.

Analytical parameters such as the limit of detection and linear dynamic range of a sensor for the determination of DA were calculated using square wave voltammetric (SWV) methods as highly sensitive electrochemical strategies. The SW voltammogram of DA at the surface of Fe_3_O_4_-SWCNTs/mim-BF_4_^−^/PE showed two linear dynamic ranges related to its concentration in the range from 0.001–10.0 µM with the Equation *I* = 1.6017 C + 1.4884 (R^2^ = 0.9903) and in the range from 10.0–220 µM with the Equation *I* = 0.2077 C + 15.9390 (R^2^ = 0.9947) (Figure 6). The Fe_3_O_4_-SWCNTs/mim-BF_4_^−^/PE showed a detection limit (Y_LOD_ = 3 S_b_/m; where S_b_ is the standard deviation of blank and m is the slope of linear dynamic range investigation) of 0.7 nM for the sensing of DA.

The selectivity of Fe_3_O_4_-SWCNTs/mim-BF_4_^−^/PE as a new sensor to determine 10.0 µM DA was checked by an acceptable error of 5% in oxidation current of the drug by the SWV method. Reported results in Table 2 confirm that Fe_3_O_4_-SWCNTs/mim-BF_4_^−^/PE could be determined DA without any important interference in real samples.

### 3.2. Real Sample Analysis of Dasatinib Anticancer Drug

In the final step and after the optimization of the sensor and investigation of kinetic and thermodynamic parameters, the ability of Fe_3_O_4_-SWCNTs/mim-BF4^−^/PE was checked to determine the dasatinib anticancer drug in real samples by the SWV method. For this purpose, dasatinib tablets and dextrose saline were selected. The standard addition method was used to analyze the DA in these samples. The results recorded in Table 3 and recovery data from 99.58% to 103.6% confirm the high-performance ability of Fe_3_O_4_-SWCNTs/mim-BF4^−^/PE as a new sensor to determine the DA in real samples.

## 4. Conclusions

The present study focused on designing and fabricating a two-fold amplified electrochemical sensor for trace-level analysis of dasatinib (an anticancer drug). For this purpose, Fe_3_O_4_-SWCNTs/mim-BF4^−^/PE was synthesized by a chemical precipitation strategy and introduced as an analytical tool. Fe_3_O_4_-SWCNTs/mim-BF4^−^/PE showed good catalytic activity of the oxidation signal of the dasatinib anticancer drug and improved its signal about 5.58 times. The suggested senor showed a high sensitivity to determine the dasatinib anticancer drug in the concentration range from 0.001–220 µM with a detection limit of 0.7 nM. In the final step, the recovery range from 99.58% to 103.6% was used to measure DA in real samples using Fe_3_O_4_-SWCNTs/mim-BF4^−^/PE as the sensor.

## Figures and Tables

**Table 1:** Comparison of the efficiency of Fe_3_O_4_-SWCNTs/1-hexyl-3-methylimidazolium tetrafluoroborate (mim-BF_4_^−^)/based on paste electrode modification (PE) with some published electrochemical sensors in the determination of dasatinib.

Electrode	Mediator	Linear Dynamic Range (µM)	Limit of Detection (µM)	Ref.
Glassy carbon	---	0.2–2.0	0.13	[70]
Pencil graphite	---	0.0092–1.0	0.0028	[42]
Carbon paste	Pt/MWCNTs and 1-butyl-3-methylimidazolium hexafluoro phosphate	5.0–500	1.0	[12]
Glassy carbon	ds-DNA + reduced graphene oxide and gold nanoparticles	0.03–5.5	0.009	[21]
Carbon paste	ZnO nanoparticle and 1-butyl-3-methylimidazolium tetrafluoroborate	1.0–1200	0.5	[71]
Carbon paste	Fe_3_O_4_-SWCNTs and mim-BF_4_^−^	0.001–220	0.0007	This work

**Table 2:** Interference study results for sensing of 10 µM dasatinib at surface of Fe_3_O_4_-SWCNTs/mim-BF_4_^−^/PE and pH = 6.0.

Species	Tolerance Limits (Weight-Substance/Weight Dasatinib)
Na^+^, Li^+^, Br^−^, CO_3_^2-^	1000
Methionine, Glycine, alanine	750
Glucose and Sucrose	650
Vitamin C, Vitamin B_2_,	400

**Table 3:** Determination of dasatinib using Fe_3_O_4_-SWCNTs/mim-BF4^−^/PE in real samples (*n* = 3).

Sample	Added (µM)	Expected (µM)	Founded (µM)	Recovery%
Tablet ^-^	2.00	2.00	2.05 ± 0.07	102.5
	10.00	12.00	11.95 ± 0.21	99.58
dextrose saline	---	---	<LOD	---
	15.00	15.00	15.54 ± 0.76	103.6
